# In vitro antimicrobial activities of Saudi honeys originating from *Ziziphus spina‐christi* L. and *Acacia gerrardii* Benth. trees

**DOI:** 10.1002/fsn3.1320

**Published:** 2019-12-16

**Authors:** Ayman A. Owayss, Khaled Elbanna, Javaid Iqbal, Hussein H. Abulreesh, Sameer R. Organji, Hael S. A. Raweh, Abdulaziz S. Alqarni

**Affiliations:** ^1^ Department of Plant Protection College of Food and Agriculture Sciences King Saud University Riyadh Saudi Arabia; ^2^ Department of Plant Protection Faculty of Agriculture Fayoum University Fayoum Egypt; ^3^ Department of Agricultural Microbiology Faculty of Agriculture Fayoum University Fayoum Egypt; ^4^ Department of Biology Faculty of Applied Science Umm Al‐Qura University Makkah Saudi Arabia; ^5^ Research Laboratories Centre Faculty of Applied Science Umm Al‐Qura University Makkah Saudi Arabia

**Keywords:** antibacterial activity, antibiotics, antifungal activity, bacteria, fungi, Sidr honey, Talh honey, zone of inhibition

## Abstract

Honeys originating from Sidr (*Ziziphus spina‐christi* L.) and Talh (*Acacia gerrardii* Benth.) trees in Saudi Arabia exhibited substantial antimicrobial activity against pathogenic gram‐positive bacteria (*Bacillus cereus*, *Staphylococcus aureus*), gram‐negative bacteria (*Escherichia coli*, *Salmonella enteritidis*), and a dermatophytic fungus (*Trichophyton mentagrophytes*). The diameter of zones of inhibition represents the level of antimicrobial potency of the honey samples. Precisely, Talh honey showed significantly higher antibacterial activity against all tested bacteria than Sidr honey. The antifungal activity of Talh and Sidr honey types was significantly at par against a dermatophytic fungus. The water‐diluted honey types (33% w/v) significantly induced a rise in the antimicrobial activity from that of the natural nondiluted honeys. Microbial strains displayed differential sensitivity; gram‐positive bacteria were more sensitive and presented larger inhibition zones than gram‐negative bacteria and the fungus. The sensitivity was highest in *B. cereus* and *S. aureus*, followed by *T. mentagrophytes*, *E. coli,* and *S. enteritidis*. The antimicrobial activity of water‐diluted honeys (Sidr and Talh) was high than that of broad‐spectrum antibacterial antibiotics (tetracycline and chloramphenicol) against bacterial strains, but these honeys were relativity less potent than antifungal antibiotics (flucoral and mycosat) against a fungal strain. Our findings indicate the antimicrobial potential of Saudi honeys to be considered in honey standards, and their therapeutic use as medical‐grade honeys needs further investigations.

## Graphical abstract

Two honey types (Sidr and Talh honey) of different floral origin in Saudi Arabia exhibited the potential antimicrobial activities against pathogenic gram‐positive bacteria (*Bacillus cereus*, *Staphylococcus aureus*), gram‐negative bacteria (*Escherichia coli*, *Salmonella enteritidis*), and a dermatophytic fungus (*Trichophyton mentagrophytes*) under laboratory conditions. Both honey types were also equally effective as tested antibiotics to control the tested microbes. The water dilution of honeys (33% w/v) significantly elevated their antimicrobial activity than natural untreated honeys. Our results support the potential use of tested honeys as therapeutic against common pathogenic microbes.

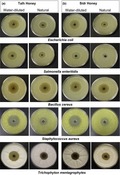

## 1 INTRODUCTION

Honey bees collect floral nectar from plants to produce natural sweet honey with a complex chemical composition. The major components of honey are simple sugars (~75% fructose and glucose), water (~20%), and sucrose (~3%–10%). Other constituents are complex sugars, minerals, vitamins, antioxidants, proteins, enzymes, phenolic compounds, and some unidentified substances (Alqarni, Owayss, & Mahmoud, 2016; Wright, Nicolson, & Shafir, 2018). In addition to its nutritional value, honey has also been used as a traditional remedy in ancient and modern cultures for curing topical burns, wounds, and numerous diseases (Abuharfeil, Al‐Oran, & Abo‐Shehada, 1999; Al‐Waili & Saloom, 1999; Eteraf‐Oskouei & Najafi, 2013; Molan, 2001; Samarghandian, Farkhondeh, & Samini, 2017). Supplementary hive products such as bee venom, royal jelly, and propolis also have potential therapeutic properties and are used in alternative medicine known as apitherapy (Basa, Belay, Tilahun, & Teshale, 2016; Pasupuleti, Sammugam, Ramesh, & Gan, 2017). The chemical composition of honey varies with the source plant of bee forage and geographical origin (Machado De‐Melo et al., 2018).

The antibacterial activity of honey was first recognized by Van Ketel in 1892 (Dustmann, 1979), which was followed by numerous studies concerning the antimicrobial properties of honey against a broad‐spectrum bacterial species (~60 species), including aerobes, anaerobes, and gram‐positive (G^+^) and gram‐negative (G^−^) bacteria (Bogdanov, 1997; Elbanna et al., 2014; Hannan et al., 2004; Kwakman & Zaat, 2012; Lusby, Coombes, & Wilkinson, 2005; Mandal & Mandal, 2011; Molan, 1992). The bactericidal and bacteriostatic potential of honey may be particularly profitable against antibiotic‐resistant bacteria (Patton, Barrett, Brennan, & Moran, 2006) and in synergizing with the antibiotic potential (Zakaria, 2015). Furthermore, honey also shows antimicrobial activity against several other microorganisms, including viruses, fungi, and yeasts (Maddocks & Jenkins, 2013; Saranraj & Sivasakthi, 2018). The development of antibiotic resistance in microorganisms attracts the use of alternative strategies such as using honey as antimicrobial agents to reduce the global load of diseases and resistance (Ayukekbong, Ntemgwa, & Atabe, 2017; S. Mandal, Pal, Chowdhury, & Debmandal, 2009; Patton et al., 2006).

In Saudi Arabia, honey consumption is gradually increasing, as honey is a principle ingredient in foods and in folk medicines (Al‐Ghamdi & Adgaba, 2015; Alqarni, 2011; Alqarni et al., 2016). Many locally produced and imported honeys are available in the Saudi market. Sidr honey and Talh honey are two major honey types in Saudi Arabia and the Arabian Peninsula. These honeys are locally named with reference to their floral nectar source. Talh honey is produced from *Acacia gerrardii* Benth. trees and Sidr honey from *Ziziphus spina‐christi L.*(Adgaba et al., 2017; Al‐Ghamdi, 2007; Al‐Khalifa & Al‐Arify, 1999; Alqarni et al., 2016). *Ziziphus* and *Acacia* are the most common plants of economic importance in Saudi Arabia and are the major floral sources of high‐valued expensive honeys (Alqarni, Hassan, & Owayss, 2015; Alqarni, 2015).

Our study aimed to evaluate the antimicrobial potential of the most preferred honeys in Saudi Arabia, Sidr and Talh honeys, against pathogenic bacterial and fungal strains. Their potential antimicrobial activity was also equated with that of antibiotics commonly used against the targeted microbial strains. This research pursuing the antimicrobial potential of honey types will be helpful in treating the pathogenic microorganisms threatening the public health and changing antibiotics into last‐resort drugs.

## 2 MATERIALS AND METHODS

### 2.1 Honey Samples

**Figure 1:**
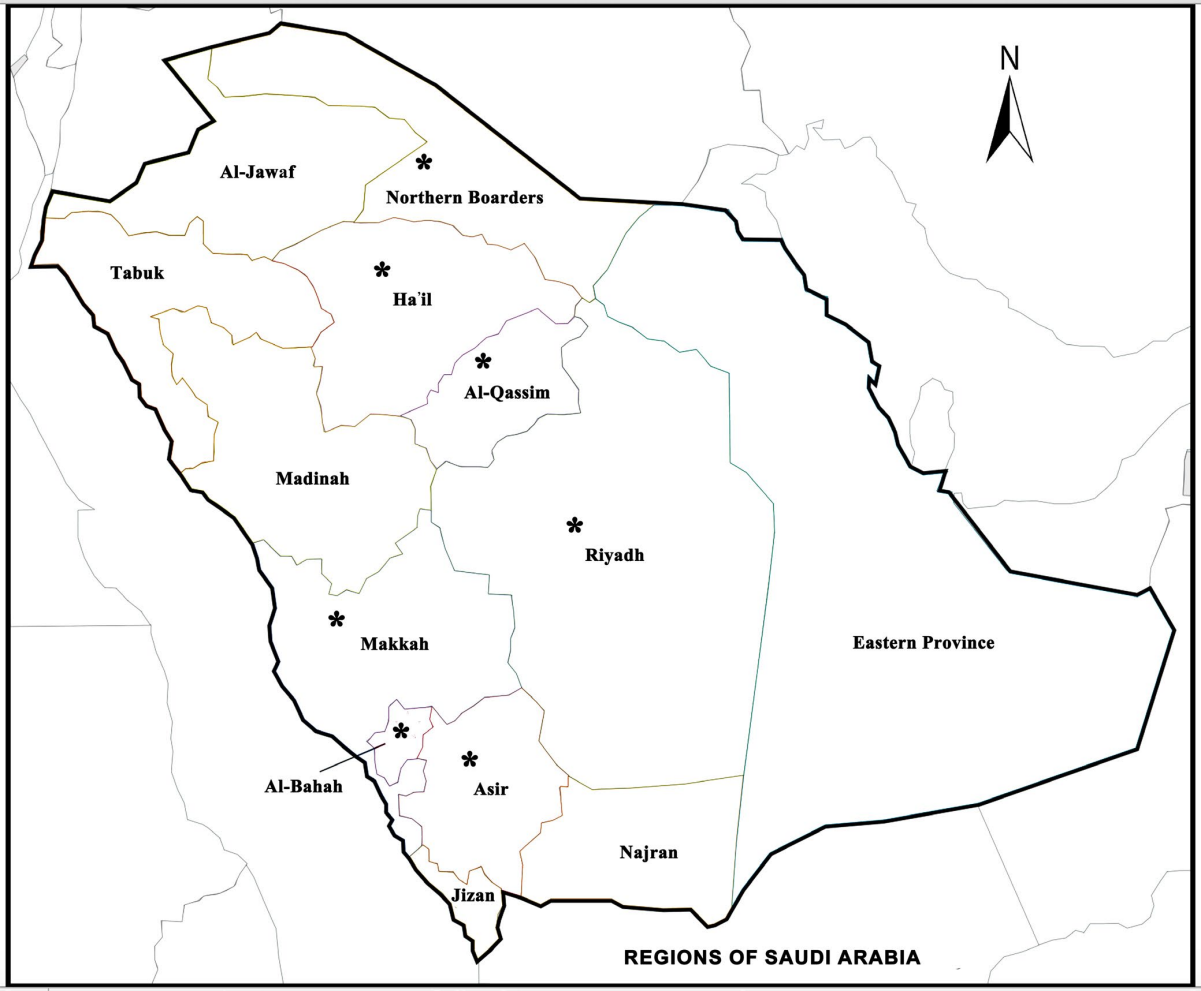
Location sites for honey collection in Saudi Arabia. Asterisks indicate the regions from where the honey samples were collected

Fresh samples (1 kg each) of the most preferred honeys for Saudi consumers, named Sidr (produced from *Z. spina‐christi* L. trees: 11 samples) and Talh (produced from *A. gerrardii* Benth. trees; 20 samples), were collected from apiaries of selected regions in the Kingdom of Saudi Arabia (KSA) (Figure 1) for in vitro analysis of their antimicrobial activities against pathogenic bacterial and fungal strains. Each honey sample from the targeted region was divided for three repeats. After running a triplicate measurement of antimicrobial activity, the mean value of these three repeats was calculated. The codes and regional data of these unifloral honeys are presented in Table 1. Two forms of honey samples, natural (nondiluted crude honey) and water‐diluted honey (33% w/v) (Elbanna et al., 2014), were used for the examination of their potential antimicrobial action.

**Table 1:** Data of Sidr (*Ziziphus spina-christi L*.) and Talh (*Acacia gerrardii Benth*.) honey samples collected from different regions of Saudi Arabia

Honey type	Botanical origin	Sample code no.	Honey source	Apiary location
Sidr honey (SH)	*Ziziphus* *spina-christi* L.	SH1	AB	Riyadh
		SH2	AB	Northern Borders
		SH3	AB	Northern Borders
		SH4	AB	Riyadh
		SH5	AB	Northern Borders
		SH6	AB	Riyadh
		SH7	AB	Riyadh
		SH8	AB	Riyadh
		SH9	AB	Riyadh
		SH10	SMA	Riyadh
		SH11	SMA	Riyadh
Talh honey (TH)	*Acacia* *gerrardii* Benth.	TH1	AB	Hail
		TH2	AB	Riyadh
		TH3	AB	Al‐Qassim
		TH4	AB	Hail
		TH5	AB	Hail
		TH6	AB	Al‐Qassim
		TH7	AB	Al‐Qassim
		TH8	AB	Hail
		TH9	AB	Hail
		TH10	AB	Riyadh
		TH11	AB	Al‐Qassim
		TH12	AB	Riyadh
		TH13	AB	Riyadh
		TH14	AB	Al‐Qassim
		TH15	RT	Al‐Baha
		TH16	RT	Assir
		TH17	RT	Makkah
		TH18	RT	Makkah
		TH19	AB	Al‐Baha
		TH20	RT	Hail

Note

AB, apiaries of beekeepers: RT, retailer; SMA, self‐monitored apiaries.

### 2.2 Microbial Strains

The microbial pathogenic strains of two gram‐positive bacteria (*Bacillus cereus* ATCC 10876 and *Staphylococcus aureus* ATCC 8095), two gram‐negative bacteria (*Escherichia coli* ATCC 25922 and *Salmonella enteritidis* ATCC 13076) and one dermatophyte fungus (*Trichophyton mentagrophytes*), were obtained from the culture collection of the Department of Biology, Faculty of Applied Sciences, Umm Al‐Qura University, Makkah, KSA. Stock cultures of bacterial and fungal strains were maintained at 4°C on nutrient and potato dextrose agar slants, respectively.

### 2.3 Assessment of Antibacterial Activity

Antimicrobial activities of each honey type (Sidr and Talh) were assessed using the well‐diffusion bioassay technique (Elbanna et al., 2014). Sterilized Muller‐Hinton or potato dextrose agar media (Oxoid) were poured into sterilized petri dishes, left to solidify at room temperature (25 ± 1°C), and swabbed with fresh bacterial or fungal strain cultures. Wells at the center of agar plates were made using a sterile cork borer (9 mm diameter) and filled with 300 µl of natural honey or water‐diluted honey (33% w/v). To give honey enough time for diffusion, all plates were placed in a refrigerator (~5°C) for 2 hr and then incubated at 37°C for 24 hr (for bacteria) and at 28°C for 48–72 hr (for the fungus). The potential antimicrobial activities of honey treatments were expressed by measuring the diameter (mm) of a clear (inhibition) zone of each well, with distilled water taken as a control. In separate experiments, the antimicrobial activity of two broad‐spectrum antibacterial (tetracycline and chloramphenicol) and two antifungal (flucoral and mycosat) antibiotics (Mast Diagnostic GmbH, Germany) were assessed against their respective microbial strains using the agar disk diffusion method and measuring the clear zone diameter (mm) of each disk (EFSA, 2012).

### 2.4 Statistical Analysis

The mean antimicrobial activity of Sidr and Talh honey samples against each tested microbial strain was measured. The data were analyzed using analysis of variance (ANOVA) under a complete randomized design after testing for homogeneity of error variances according to the procedure defined by Gomez and Gomez (1984). InfoStat software (Rienzo et al., 2016) was used for the statistical analysis. Statistical means were compared for significant differences at *p* ≤ .05 using the least significant difference (LSD) test.

## 3 RESULTS

### 3.1 Antimicrobial Activity of Honeys

In vitro antimicrobial activities of the most common unifloral honey types in Saudi Arabia (Sidr honey (SH) and Talh honey (TH) were evaluated against pathogenic strains of gram‐positive bacteria (*B. cereus, S. aureus*), gram‐negative bacteria (*E. coli, S. enteritidis*), and dermatophyte fungi (*T. mentagrophytes*). Natural and water‐diluted (33% w/v) forms of SH and TH were used for testing their potential antimicrobial activity. The data revealed that SH and TH honey types have significant differential antimicrobial potentialities against the tested microbial strains. The microbial strains were significantly inhibited as measured in terms of their zone of inhibition (ZOI), and a large ZOI reflects a high sensitivity of tested microbial strains. No microbial strains were resistant to any of the honey types.

The microbial strains presented differential sensitivity to the honey types. Gram‐positive (G^+^) bacteria were more sensitive to both honey types (SH and TH), with significantly higher ZOI values than those of gram‐positive (G^‐^) bacteria and fungi (Table 2). *B. cereus* (G^+^) showed the greatest inhibition (largest ZOI) by SH (31.09 ± 0.84 mm and 36.45 ± 1.01 mm: natural and water‐diluted, respectively) and TH (35.65 ± 0.53 mm and 41.65 ± 0.68 mm: natural and water‐diluted, respectively). *S. aureus* (G^+^) presented the second most inhibition (ZOI) by SH (29.45 ± 0.73 and 34.55 ± 1.08 mm: natural and water‐diluted, respectively) and TH (32.00 ± 0.61 and 37.70 ± 0.70 mm: natural and water‐diluted, respectively). The least inhibition (smallest ZOI) was recorded for *S. enteritidis* (G^‐^ bacteria) with SH (19.36 ± 0.64 and 23.36 ± 0.79 mm: natural and water‐diluted, respectively) and TH (23.35 ± 0.53 and 28.10 ± 0.67 mm: natural and water‐diluted, respectively). The descending sensitivity order was *B. cereus > S. aureus *> *T. mentagrophytes *> *E. coli > S. enteritidis* (Table 2)*.* Extraordinarily, these measured ZOIs of microbial strains remained unchanged when plates were left for more than ten days, and no microbial growth occurred when new agar plates or broth media were inoculated with a loop sampling the clear zone, suggesting that both tested honey types (SH and TH) have a lethal bactericidal effect. The dermatophyte fungus (*T. mentagrophytes*) was equally sensitive to both honey types, natural honey (25.91 ± 063 and 25.75 ± 0.62 mm: SH and TH, respectively) and water‐diluted honey (30.73 ± 0.98 and 30.85 ± 0.78 mm: SH and TH, respectively).

**Table 2:** Diameter of inhibition zone indicating the antimicrobial activity of Sidr and Talh honey samples against pathogenic gram‐positive (G^+^) bacteria, gram‐negative (G^‐^) bacteria, and a dermatophyte fungus

Microbial strain	Zone of inhibition (ZOI) in mm ± *SEM*			
	Sidr Honey (SH)		Talh Honey (TH)	
	Natural	Water‐diluted (33% w/v)	Natural	Water‐diluted (33% w/v)
G^+^ Bacteria				
*Bacillus cereus*	31.09 ± 0.84^a^	36.45 ± 1.01^a^	35.65 ± 0.53^a^	41.65 ± 0.68^a^
*Staphylococcus aureus*	29.45 ± 0.73^a^	34.55 ± 1.08^a^	32.00 ± 0.61^b^	37.70 ± 0.70^b^
G^−^ Bacteria				
*Escherichia coli*	23.18 ± 0.83^c^	27.09 ± 1.05^c^	27.15 ± 0.67^c^	31.20 ± 0.78^c^
*Salmonella enteritidis*	19.36 ± 0.64^d^	23.36 ± 0.79^d^	23.35 ± 0.53^e^	28.10 ± 0.67^c^
Fungus				
*Trichophyton mentagrophytes*	25.91 ± 0.63^b^	30.73 ± 0.98^b^	25.75 ± 0.62^d^	30.85 ± 0.78^d^

Note

With the largest ZOIs, gram‐positive bacteria are more sensitive to Sidr and Talh honey than the other microbes. Means with common letters are not significantly different (*p* ≤ .05) as analyzed by the ANOVA followed by the least significant difference (LSD) test. SEM: Standard error of mean.

**Figure 2:**
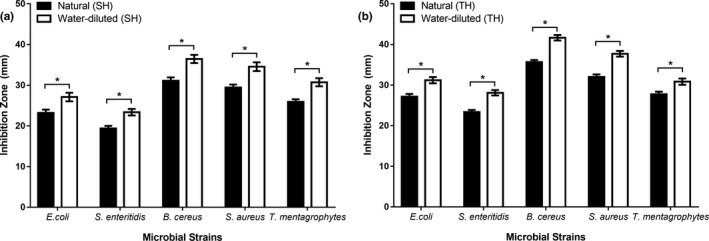
Comparison of the antimicrobial activity of natural versus diluted honeys: (a) Sidr (b) Talh. Water‐diluted honey has significantly higher antimicrobial activity than natural honey. The asterisks indicate the significant difference between the graph bars

Antimicrobial activities of honeys were significantly amplified when natural honeys were diluted with water (33% w/v). A comparison of the antimicrobial activities of individual honey types, that is, natural SH versus water‐diluted SH (Figure 2a) and natural TH versus water‐diluted TH (Figure 2b) showed significantly higher inhibition in water‐diluted honeys against all tested G^+^ and G^‐^ bacterial strains, and fungal strains.

**Figure 3:**
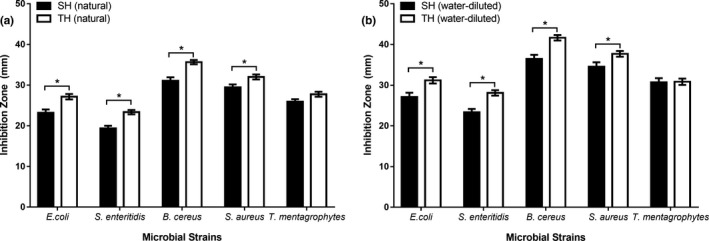
Comparison of the antimicrobial activity of Sidr and Talh honeys: (a) natural honey (b) water‐diluted honey. Talh honeys have higher antimicrobial activity than Sidr honey. The asterisks indicate the significant difference between the graph bars

**Figure 4:**
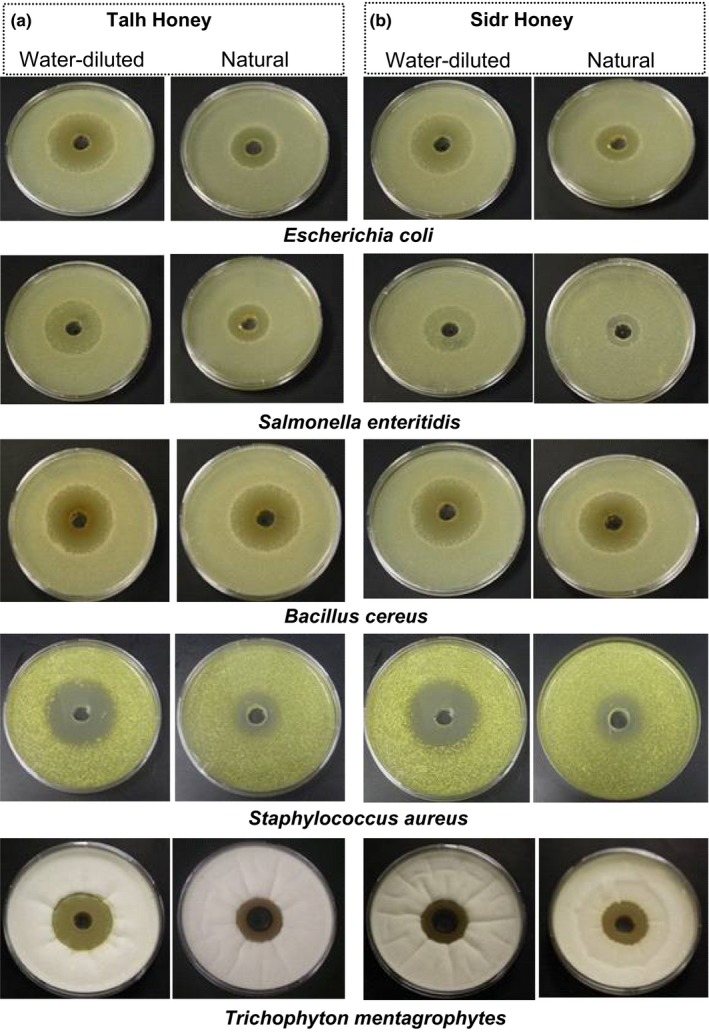
The zone of microbial growth inhibition on the cultures of bacteria and dermatophyte fungus obtained after adding natural and water‐diluted honeys: (a) Talh honey and (b) Sidr Honey

TH displayed higher antimicrobial activity than SH against G^+^ and G^‐^ bacteria but not against the fungal strain, where both honey types were significantly similar (Figure 3). The comparison of the antimicrobial activities between natural SH and natural TH (Figure 3a), and between water‐diluted SH and water‐diluted TH (Figure 3b) revealed that each form of TH was more effective than the respective form of SH against a single microbial strain. Figure 4 displayed the antimicrobial activity of the tested honeys with zone of microbial growth inhibition on the cultures of tested microbial strains.

### 3.2 Antimicrobial Activity of Antibiotics

The disk diffusion test for antibiotics evaluated the antimicrobial activity of two antibacterial (tetracycline and chloramphenicol) and two antifungal (flucoral and mycosat) antibiotics against their respective microbial strains. Our results indicated a significant difference among the antimicrobial activities of the tested antibiotics.

**Figure 5:**
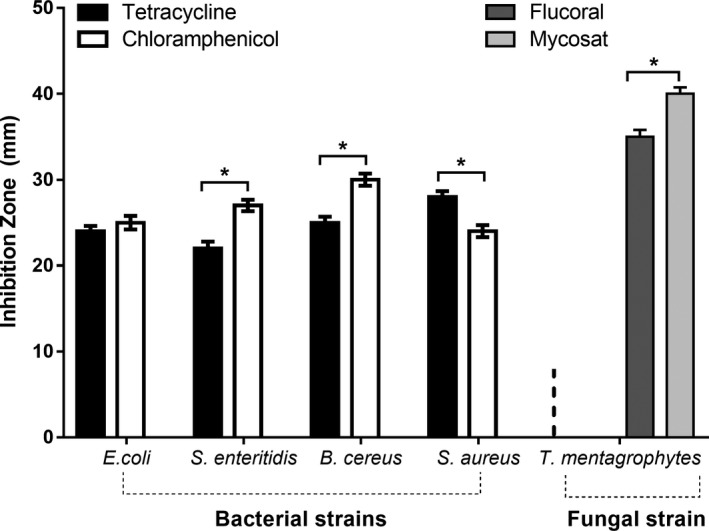
Comparison between antibiotics for their antimicrobial activity against single microbial strains. The asterisks indicate the significant differences between the antibiotics

For antibacterial antibiotics, the largest ZOI was recorded for *S. aureus* (G^+^) against tetracycline (28.00 ± 0.67 mm) and for *B. cereus* (G^+^) against chloramphenicol (30.00 ± 0.71 mm). *S. enteritidis* exhibited the smallest ZOI (22 ± 0.79 mm) with tetracycline, whereas *S. aureus* showed the smallest ZOI (24 ± 0.70 mm) for chloramphenicol (Table 3). For antifungal antibiotics, mycosat was relatively more effective, having the largest ZOI (40.00 ± 0.75 mm), than flucoral (35.00 ± 0.79 mm) against the fungus *T. mentagrophytes* (Table 4). Of the antibacterial antibiotics, chloramphenicol was significantly more potent against *B. cereus* and *S. enteritidis*, and tetracycline was significantly more potent against *S. aureus*. However, the antimicrobial effects of these two antibiotics were significantly similar against *E. coli.* Of the antifungal antibiotics, mycosat showed significantly higher antimicrobial action against *T. mentagrophytes* than flucoral (Figure 5).

**Table 3:** Antimicrobial activities of antibacterial antibiotics against mycobacterial strains

Microbial strain	Diameter (mm) of inhibition zone ± *SEM* *	
	Antibacterial antibiotics	
	Tetracycline (30 µg/ml)	Chloramphenicol (30 µg/ml)
G^+^ Bacteria		
*Bacillus cereus*	25 ± 0.68^b^	30 ± 0.71^a^
*Staphylococcus aureus*	28 ± 0.67^a^	24 ± 0.70^c^
G^−^ Bacteria		
*Escherichia coli*	24 ± 0.62^b^	25 ± 0.78^b,c^
*Salmonella enteritidis*	22 ± 0.79^c^	27 ± 0.67^b^

* Well‐diffusion assay. Means with the common letters within the same column are not significantly different from each other (*p* ≤ .05) as analyzed by the ANOVA followed by the least significant difference (LSD) test. SEM: Standard error of mean.

**Table 4:** Antimicrobial activities of antifungal antibiotics against the fungal strain

Antibiotics (Antifungal)	Diameter (mm) of inhibition zone ± *SEM* *
	Fungal strain
	Trichophyton mentagrophytes
Flucoral (100 μg/ml)	35.00 ± 0.79^b^
Mycosat (100 μg/ml)	40.00 ± 0.75^a^

* Disk diffusion assay. Means with the common letters within the same column are not significantly different from each other (*p* ≤ .05) as analyzed by the ANOVA followed by the least significant difference (LSD) test. SEM: Standard error of mean

### 3.3 Comparison Among Antimicrobial Action of Honey and Antibiotics

**Figure 6:**
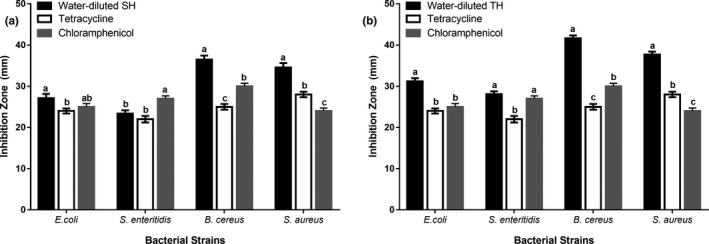
Comparison of antimicrobial activities of antibacterial antibiotics with water‐diluted SH (a) and water‐diluted TH (b). The common letters on bars indicate no significant difference

**Figure 7:**
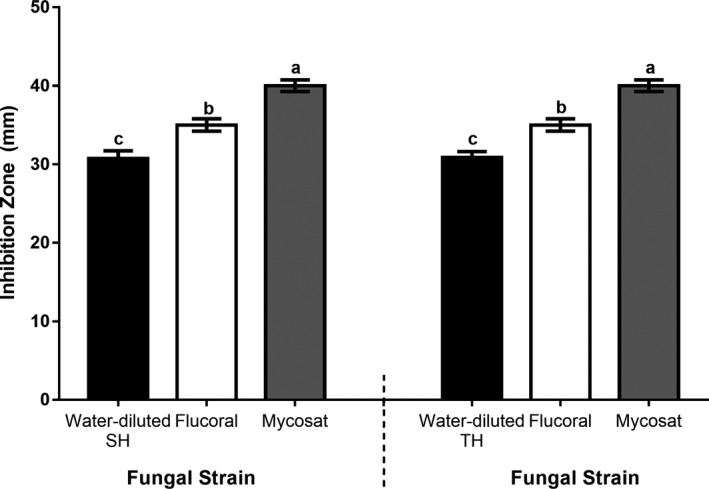
Comparison between antifungal antibiotics and water‐diluted honeys (SH and TH) for their antimicrobial activity against *Trichophyton mentagrophytes*. The common letters on the bars indicate no significant difference

It is apparent from the data analysis that the high antimicrobial activity (larger ZOIs) shown by bacterial strains particularly with water‐diluted SH (Figure 6a) and water‐diluted TH (Figure 6b) is significantly greater than that of the tested broad‐spectrum antibacterial antibiotics (tetracycline and chloramphenicol). *S. enteritidis *(gram‐negative bacteria) treated with water‐diluted SH showed exception where ZOIs values were significantly lower than chloramphenicol but significantly at par with tetracycline (Figure 6a). Nevertheless, antifungal antibiotics exhibited significantly higher antimicrobial activity against the fungal strain than the tested water‐diluted SH and TH honeys (Figure 7).

## 4 DISCUSSION

### 4.1 Antimicrobial activity of honeys

Saudi Sidr honey (SH) and Talh honey (TH) displayed substantial antimicrobial activities against tested pathogenic microbial strains. These primary findings strengthened the idea for using Saudi honeys as potential alternative broad‐spectrum strategy to treat bacterial and fungal infections. Use of various types of honeys due to its antimicrobial effects has been published in numerous studies (Bradshaw, 2011; Israili, 2014; McLoone, Warnock, & Fyfe, 2016). However, more extensive research is necessary for conclusive declaration as substituting broad‐spectrum antimicrobial drugs with these Saudi honeys. Some previous studies described the physiochemical properties of honeys and compared the antimicrobial action of various Saudi honey types with those of imported honeys in different experimental setups (Al‐Nahari et al., 2015; Alqurashi, Masoud, & Alamin, 2013; Hegazi & Abd Allah, 2012). However, the present study compared the two most preferred local Saudi honeys for their antimicrobial potential against bacterial and fungal strains. It is concluded that TH possesses higher antimicrobial activity against bacterial strains than SH. These findings are confirmed by the noticeable higher acidity and total phenolic contents in TH than in SH (Alqarni et al., 2016). The phenolic contents in honey are directly connected with increased antibacterial activities (Alvarez‐Suarez et al., 2010; Stagos et al., 2018). Another possible factor for the substantial antimicrobial activity of honey is probably the synergism between H_2_O_2_ and phenolic compounds that exert a pro‐oxidant activity that may lead to the degradation plasmidic DNA (Poli et al., 2018). The difference in floral origin of tested honey types could be another potential dominant reason for their differential antimicrobial activities (Allen, Molan, & Reid, 1991; Elbanna et al., 2014; Willix, Molan, & Harfoot, 1992). In addition, the geographical location and seasonality could also influence the antimicrobial activity of different honey types (Al‐Waili, Salom, Butler, & Al Ghamdi, 2011; Molan & Cooper, 2000). A previous study reported that SH had higher antibacterial activity than Somur and Meria honeys (Al‐Haik, Al‐Haddad, Al‐kaf, Hassan, & Edrees, 2018). In contrast, our study illustrated another honey (TH) exhibiting superior antibacterial activity against selected bacterial strains compared to SH.

The diameter for the zone of inhibition (ZOI) indicates the sensitivity of microbial strains. All recorded diameters of the ZOIs in the present study were greater than 11 mm. This result aligns well with the declaration of Agbagwa and Frank‐Peterside (2010) that “the diameter of inhibition zones less than 7 mm corresponds to resistant microorganisms and greater than 11 mm suggests that the microorganisms are sensitive to antimicrobial agent.” Thus, our findings are consistent in that all tested microbial strains were sensitive to tested honeys, and these honeys are proposed as prospective antimicrobial agents to benefit human health.

Both SH and TH showed broad‐spectrum antimicrobial potential against G^+^ and G^‐^ bacteria and fungi, which is consistent with previous findings in which different honey types of diverse floral origins were reported with broad‐spectrum activity against G^+^ and G^‐^ bacteria (Almasaudi et al., 2017; Al‐Naama, 2009; Elbanna et al., 2014; Irish, Blair, & Carter, 2011; Lusby, Coombes, & Wilkinson, 2002; Radwan, El‐Essawy, & Sarhan, 1984). The microbial strains presented differential sensitivity to the honey types: G^+^ bacteria were more sensitive than G^‐^ bacteria and fungi. Hegazi and Abd Allah (2012) reported Saudi honeys (20.30%) from twelve different floral sources (including Sidr) as effective antibacterial agents against G^+^ (*S. aureus*, *Streptococcus pyogenes*, *Corynebacterium pseudotuberculosis*) and G^−^ (*Klebsiella pneumoniae, Pseudomonas aeruginosa,* and *E. coli*) bacterial pathogens. These honeys were less effective against *E. coli* than the other bacteria and contradict our findings in which SH and TH were significantly effective against *E. coli*, similar to other tested microbial strains. In partial confirmation, Saudi Sidr honey was found to be more efficient than mountain honey against G^‐^ bacteria (*E. coli, K. pneumoniae, P. aeruginosa* and *A. baumannii*), with a high sensitivity of *E. coli* toward Sidr honey (Alqurashi et al., 2013). Saudi honeys named Shaoka (*Fagonia cretica*) and Taify Sidr (*Z. spina‐christi*) were more potent than Manuka honey (*Leptospermum scoparium*) against single G^‐^ bacteria (*S.* *enteritidis*) in terms of ZOI equivalents in phenol percentages (7.3%, 8.4%, and 6.9%), respectively, and antimicrobial activity was independent of the honey color (Halawani & Shohayeb, 2011).

SH and TH presented lethal bactericidal and fungicidal effects because no further change in the inhibition zone was detected even after ten days. Al‐Nahari et al. (2015) evaluated that the antimicrobial effect of Manuka honey (*L. scoparium*) was more evident than that of Seder and *Nigella sativa* honey against both antibiotic (imipenem)‐resistant and antibiotic‐sensitive bacteria (*P. aeruginosa*). Manuka honey was bactericidal, but Seder and *N. sativa* honeys were only bacteriostatic. In contrast, SH was completely bactericidal against our tested bacterial strains.

Saudi honeys showed dose‐dependent antibacterial activity: Sidr (*Z. spina‐christi*) and Dharm (*Lavandula dentata*) were more potent at high concentrations (50%–80% w/v) against *E. coli*, *Proteus mirabilis*, *S. aureus*, *Shigella flexneri*, and *S. epidermidis* than Majra honey (*Hypoestes forskaolii*) (Ghramh, Khan, & Alshehri, 2018). In contrast, only one concentration of water‐diluted honey (33% w/v) was adopted from Elbanna et al. (2014) and substantially inhibited the tested microbial strains. Exploring the antimicrobial activity with a series of honey dilutions could be a potential future investigation to determine the dose dependency (if any).

SH and TH honeys also demonstrated equal fungicidal potential against a dermatophytic fungus (*T. mentagrophytes*) with high inhibition. This is in line with previous studies regarding the antifungal action of other honey types (Manuka, Medihoney, Nigerian, etc.) for some yeasts and fungi, such as *Aspergillus*, *Penicillium*, *Candida,* and common dermatophytes (Anyanwu, 2012; Brady, Molan, & Harfoot, 1996; Carter, Blair, Irish, & Shokohi, 2006). Conversely, fungi (*Aspergillus nidulans*) were less sensitive to honey samples, including Talh and Sidr, than bacteria (Al‐Waili et al., 2013).

Water‐diluted (33% w/v) honeys revealed an elevated antimicrobial activity as compared to nondiluted honeys. An enzymatic reaction of glucose oxidase is being active in water–honey medium. Hydrogen peroxide is produced when glucose oxidase oxidizes glucose to gluconic acid (Mandal & Mandal, 2011). Synthesis of hydrogen peroxide in water‐diluted honeys could be the potential reason for elevated antimicrobial activity. This also explains why nectar (in plant or in bee stomach or in unripe honey) is not infected with microbes. The dilutions of honey between 30% and 50% (v/v) led to maximum levels of accumulated hydrogen peroxide (Bang, Buntting, & Molan, 2003), and the dilution range was similar to our tested honey dilution concentrations (33% w/v). However, the antimicrobial activity of honey is extremely complex and might be due to the involvement of multiple compounds and several nonperoxide components that are also reported to contribute to the unique antibacterial activity of honey, such as physico‐chemical properties, osmotic pressure, acidic pH, and nonperoxide phytochemical components, including antioxidants and antimicrobial peptides (Ayaad, Shaker, & Almuhnaa, 2009; Brudzynski, 2006; Halawani & Shohayeb, 2011; Kwakman & Zaat, 2012; Mavric, Wittmann, Barth, & Henle, 2008; Molan, 1992; Molan & Russell, 1988; Simon et al., 2009). Elbanna et al. (2014) attributed the antimicrobial activity of three unifloral Egyptian honeys (~88%) to nonperoxide agents, whereas hydrogen peroxide contributed less (~12%) to the tested honeys. In contrast, some scientists reported a fourfold decline in the antimicrobial activity of honey upon dilution (Adeleke, Onakoya, & Alli, 2002; Olaitan, Adeleke, & Ola, 2007), possibly due the presence of catalase in water that neutralized the hydrogen peroxide (Szweda, 2017). Due to the presence of numerous compounds in honey, bacterial resistance is less likely to be developed in honey‐treated bacteria (Carnwath, Graham, Reynolds, & Pollock, 2014; Machado De‐Melo et al., 2018), favoring the use of honeys against microbial infections.

### 4.2 Antimicrobial activity of antibiotics

In the present study, broad‐spectrum antibacterial (tetracycline and chloramphenicol) and antifungal (flucoral and mycosat) antibiotics were also effective against their respective microbes. Interestingly, the antibacterial activity of water‐diluted SH and TH was greater than that of the tested antibacterial antibiotics. These findings should be considered as indicative rather than conclusive, as varied doses and two different testing methods were used for evaluation of antimicrobial activity. Karayil, Deshpande, and Koppikar (1998) and Elbanna et al. (2014) found that water‐diluted honey inhibited the growth of certain pathogenic bacteria relatively more than some antibiotics. Although the tested antibiotics and bacterial strains were different from those in our study, the elevated effectiveness of water‐diluted honey over tested antibiotics is in consistent with our findings. Agbagwa and Frank‐Peterside (2010) found better antibacterial activity for SH than for imipenem (antibiotic) against a pathogenic G^‐^ bacterium (*P. aeruginosa*) and partially supported our results regarding superior antibacterial activity of SH compared with tested antibiotics. Nigerian honey samples (40% v/v) gave better antimicrobial activity against *P. aeruginosa, S. aureus, E. coli,* and *K. pneumoniae* than four antibiotics, namely amoxicillin, streptomycin, ceftriaxone, and erythromycin (Braide et al., 2012). Based on the published reports in literature (Israili, 2014; Liu et al., 2018), it is likely predictable that the use of honey in combination with antibiotics could synergize the antimicrobial activity. Müller et al. (2013) found a synergistic effect between Medihoney and rifampicin antibiotic on *S. aureus* but not between Manuka honey and rifampicin. Thus, further investigations with different honeys and common broad‐spectrum antibiotics may unveil their synergism against microbes to establish their parallel use as an effective antimicrobial therapy.

### 4.3 Honey as a promising therapeutic alternative to antimicrobial agents

Honey is traditionally used as therapeutic agent against skin infections and wounds caused by microbial pathogens (Israili, 2014; Liu et al., 2014; McLoone et al., 2016). Our results presented the potent antimicrobial prosperities of SH and TH against skin infection causing bacterial agents and dermatologically important filamentous fungi. These findings suggest the prospective use of Saudi honeys in the clinical treatments of different microbial infections. The antimicrobial activity of honey could be due to its various contents such as high sugar, total phenolic compounds and hydrogen peroxide levels. Furthermore, the bactericidal mechanisms of these content may include DNA degrading activity, interruption of cell division, alteration in the cell morphology and general loss of structural integrity of the microbial cell (Israili, 2014; Johnston, McBride, Dahiya, Owusu, & Nigam, 2018). The microbes may not develop resistance against honey in the same way as they develop for other commonly used antimicrobial agents. These features may make the honey a promising alternative to the commonly used antibiotics. 

## 5 CONCLUSION

Conclusively, Sidr and Talh honey samples have significant antimicrobial potential against gram‐positive and gram‐negative bacteria and dermatophytic fungi regardless of the sample origin. Talh honey was more potent against tested microbial strains than Sidr honey. Water dilution of honeys elevated the antimicrobial activity above that of natural nondiluted honeys. Microbial strains showed differential sensitivity, and G^+^ bacteria were more sensitive than G^‐^ bacteria and fungi. The in vitro antimicrobial activity of honeys was comparable with that of common broad‐spectrum antibacterial antibiotics. Our findings are indicative of the potential antimicrobial quality of Saudi honeys considered in honey standards, and further investigations are necessary to standardize the Sidr and Talh honeys for their therapeutic applications as medical‐grade honeys.

## CONFLICT OF INTEREST

The authors declare that they do not have any conflict of interest.

## AUTHOR CONTRIBUTIONS

Ayman A. Owayss and Abdulaziz S. Alqarni designed the field experiments and executed them with the contributions of Javaid Iqbal and Hael S.A. Raweh. Khaled Elbanna, Hussein H. Abulreesh and Sameer R. Organji planned and conceived the laboratory tests. Ayman A. Owayss. and Khaled Elbanna arranged the data and wrote the preliminary manuscript. Javaid Iqbal analyzed the results, constructed the graphs and revised the manuscript. All authors reviewed and approved the final version of the manuscript.

## ETHICAL STATEMENT

This study does not involve any human or animal testing. Informed Consent: Consent was obtained from all study participants for its submission in this journal.
